# Long-term consumption of sugar-sweetened beverage during the growth period promotes social aggression in adult mice with proinflammatory responses in the brain

**DOI:** 10.1038/srep45693

**Published:** 2017-04-10

**Authors:** Jung-Yun Choi, Mi-Na Park, Chong-Su Kim, Young-Kwan Lee, Eun Young Choi, Woo Young Chun, Dong-Mi Shin

**Affiliations:** 1 Department of Food and Nutrition, Seoul National University, Seoul 08826, Korea; 2 Department of Biomedical Sciences, Graduate School of Seoul National University, Seoul 03080, Korea; 3 Department of Psychology, Chungnam National University, Daejeon 34134, Korea; 4 Research Institution of Human Ecology, Seoul National University, Seoul 08826, Korea

## Abstract

Overconsumption of sugar-sweetened beverages (SSBs) is known to be a key contributor to the obesity epidemic; however, its effects on behavioral changes are yet to be fully studied. In the present study, we examined the long-term effects of SSB on social aggression in mice. Three-week-old weaned mice started to drink either a 30 w/v% sucrose solution (S30), plain water (CT), or an aspartame solution with sweetness equivalent to the sucrose solution (A30) and continued to drink until they were 11-week-old adults. Aggressive behaviors were assessed by the resident-intruder test. We found that SSB significantly promoted social aggression, accompanied by heightened serum corticosterone and reduced body weight. To understand the underlying mechanism, we performed transcriptome analyses of brain. The profiles of mice on S30 were dramatically different from those on CT or A30. Transcriptional networks related to immunological function were significantly dysregulated by SSB. FACS analysis of mice on S30 revealed increased numbers of inflammatory cells in peripheral blood. Interestingly, the artificial sweetener failed to mimic the effects of sugar on social aggression and inflammatory responses. These results demonstrate that SSB promotes aggressive behaviors and provide evidence that sugar reduction strategies may be useful in efforts to prevent social aggression.

Aggressive behavior has been traditionally defined as an overt behavior with the intention of inflicting damage on another individual^1^. The potential for aggressive behavior exists whenever the interests of two or more individuals conflict with broad similarities across species from mice to man^1^. Although aggression has advantages in competitive situations for obtaining food or defending territories and mates from competitors in the wild animal kingdom^2^, it is considered as one of the major social problems in human society. The World Health Assembly officially declared violence as a major public health issue in 1996, and the World Health Organization (WHO) released the first World Report on Violence and Health in ref. 3. In addition, various forms of violence have incurred huge costs in treating victims and repairing infrastructure. Therefore, it is important to identify relevant factors to promote or suppress aggressive behavior for effective interventions, thereby improving public health.

Intrinsic factors that modulate aggressive behaviors may include neuropeptides and neurotransmitters, which are small protein-like molecules, or chemical messengers regulating neuronal signaling. Vasopressin and oxytocin are frequently identified as neuropeptides related to aggressive behaviors. Several animal studies have demonstrated that oxytocin can reduce aggression^4^,^5^, albeit with other conflicting results^6^, while vasopressin is reported to increase male-male aggression in birds and mammals^7^,^8^. In addition, a glucocorticoid hormone, cortisol in human and corticosterone in rodents, is widely studied as a stress hormone and a well-established target in the search for hormonal modulators of social aggression^9^,^10^. In addition to the roles of cortisol in temper and behaviors, it orchestrates a range of different metabolic processes. Cortisol is known to promote catabolic changes in the body, therefore, reduction in body weight driven by heightened cortisol is often linked to aggressive behaviors in human and animal model studies^11^,^12^,^13^,^14^. Lower levels of serotonin, a neurotransmitter related to mood, were reported to induce impulsivity and aggression^15^. Recently, transcript levels of genes such as *Mecp2, Adrbk2, and Maoa* and a few transcriptional signatures including NF-kB and MAPK signaling have been studied in relation to aggressive behavior^16^,^17^,^18^. Furthermore, environmental factors including socioeconomic status and lifestyles have also been shown to affect aggressive behaviors^11^. Among such extrinsic factors, diet, one of the daily necessities of life, is believed to determine spirituality, mental well-being, intelligence, and temperament, including antisocial or aggressive behavior^19^. For instances, administration of tyrosine, an amino acid serving as precursor of catecholamines and neurotransmitters, such as adrenaline and dopamine, was reported to lead to a significant increase in global mood scores in human^20^. Tryptophan-rich diet was also reported to increase the level of serotonin and thereby elevate the mood in older women^21^. With regard to cognitive behavior and mood, caffeine enhanced executive control and working memory with reduced feelings of fatigue^22^. Similarly, sugar has recently drawn attention due to possible roles in modulating behavior and mood as well as health and disease.

Sugars are found in the diet either as a natural component of food or as an additive to foods and beverages. Although added sugar enhances food desirability by sweetening foods and beverages, it provides only empty calories with no nutrient value^23^. Considering potential negative effects of excessive sugar intake, the WHO has recently suggested recommendations on the intake of free sugars in adults and children, with a particular focus on the prevention and control of unhealthy weight gain and dental caries^24^. Increase in sugar intake could be attributable to rising consumption of sugar-sweetened beverages (SSBs)^25^,^26^. Numerous recent studies have reported that overconsumption of SSBs might be related to metabolic diseases such as obesity, type 2 diabetes and even cancers^27^,^28^,^29^,^30^,^31^,^32^. While overconsumption of SSBs has been extensively studied for associations with physical health problems, its effects on behavioral changes have not been fully studied.

The association between aggressive behavior and sugar consumption remains controversial and inconsistent. One study reported that a high level of sugar consumption was positively related with destructive-aggressive behaviors in hyperactive children^33^. Others found that reduced sugar intake might be linked to a lower incidence of formal disciplinary actions in juvenile prison inmates^34^,^35^,^36^. Furthermore, in adolescents, a relationship between levels of consumption of sugar-containing soft drinks and behavioral problems was found in a cross-sectional population-based survey^37^. In contrast, no such correlation was found in a study, in which oppositional or aggressive behaviors were assessed in the school-aged children provided with a sugar-sweetened diet^38^. A lack of systematic animal studies to examine the causal relationship of sugar consumption with behavior problems is partly attributable to the as yet incomplete understanding of the effects of sugar intake on aggression.

In the present study, we aimed to investigate the effects of sugar on social aggression in an animal model. We assessed aggressive behaviors in mice that consumed a high sucrose solution from infancy to adulthood, with those given an artificial sweetener solution or plain water. The levels of stress and aggression-related hormones were determined along with body weight changes. In addition, to elucidate the underlying molecular mechanisms of aggressive behaviors induced by sucrose consumption, we performed transcriptome analyses of brain tissues. Findings from the transcriptome analysis prompted us to assess changes in numbers of inflammatory cells in peripheral blood using flow cytometry. The results show that continuous over-consumption of sucrose solution from infancy to adulthood leads to increased social aggression with pro-inflammatory responses in the brain. In addition, our findings provide insights into understanding the physiological and molecular mechanisms by which sugar plays roles in promoting aggressive behaviors.

## Results

### Sucrose consumption led to a significant increase in corticosterone levels

**Figure 1:**
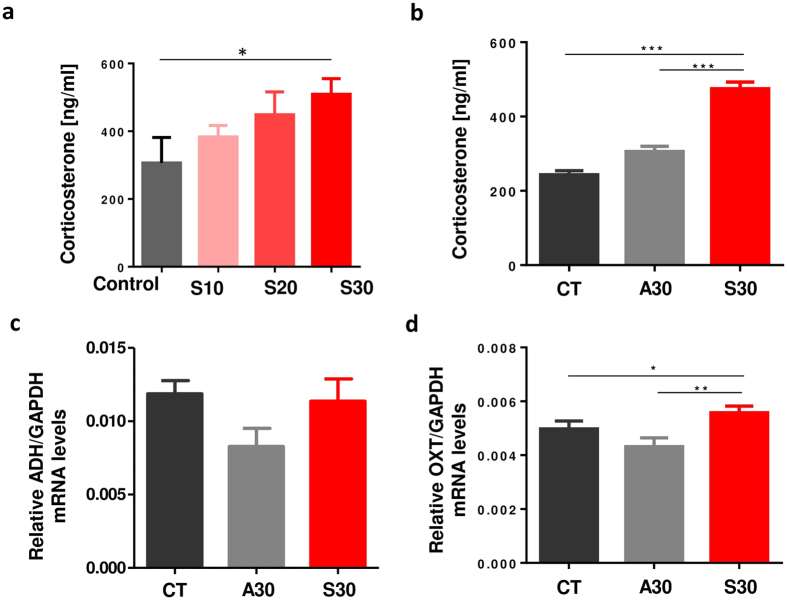
Levels of corticosterone, vasopressin and oxytocin in mice. (**a**) Levels of corticosterone in 6-week old mice (4 mice per each group) that consumed plain water, 10 w/v%, 20 w/v% or 30 w/v% sucrose solution for 2 weeks were determined by ELISA. (**b**) Levels of serum corticosterone following consumption of sucrose solution, aspartame solution, or plain water for 8-week (8–9 mice per each group). (**c**,**d**) Relative mRNA expression level of ADH (**c**) and oxytocin (**d**) in mice following consumption of plain water, sucrose solution or aspartame solution for 8-week were determined by qPCR. The expression of each gene was normalized to GAPDH. All data are presented as mean ± S.E.M. Mann-Whitney U test was performed to determine significant difference in S30 compared with CT or A30. *p < 0.05, **p < 0.01, ***p < 0.001.

Since corticosterone secretion was previously reported to be related to aggressive behavior^10^, we first asked if serum corticosterone levels could be affected by sucrose consumption over a short-term period. Similar to cortisol in humans, corticosterone is a glucocorticoid secreted from the adrenal cortex in rodents. Corticosterone levels in the blood were elevated in a dose-dependent manner in mice that consumed sucrose solutions for 2 weeks compared to control mice consuming plain water (Fig. 1a). We found significant increases in corticosterone levels only in mice that consumed the highest concentration of sucrose. This result prompted us to investigate the long-term effects of the high-dose sucrose solution. 3-week old weaned C57BL/6J male mice were provided with sucrose solution *ad libitum* until 11 weeks of age (S30). Consistent with previous results from a short-term experiment, the levels of serum corticosterone in S30 mice were significantly higher than for control mice that consumed plain water (CT). Interestingly, a significant difference was also found in the comparison of mice on S30 and those mice that consumed an artificial sweetener (aspartame) solution with the sweetness equivalent to the S30 sucrose solution (A30) (Fig. 1b). Other hormones known to be related to aggressive behavior were investigated as well. The transcriptional levels of vasopressin (ADH) did not differ among the groups (Fig. 1c). However, S30 mice showed a significant increase in oxytocin (OXT) compared to CT or A30 mice (Fig. 1d). These findings implied that continuous consumption of a sugar solution from infancy to adulthood may lead to hormonal changes that might be related to the development of aggressive behaviors and that the hormonal changes are not due to sweet taste alone.

### Long-term consumption of sucrose solution elicited aggressive behaviors in mice

**Figure 2:**
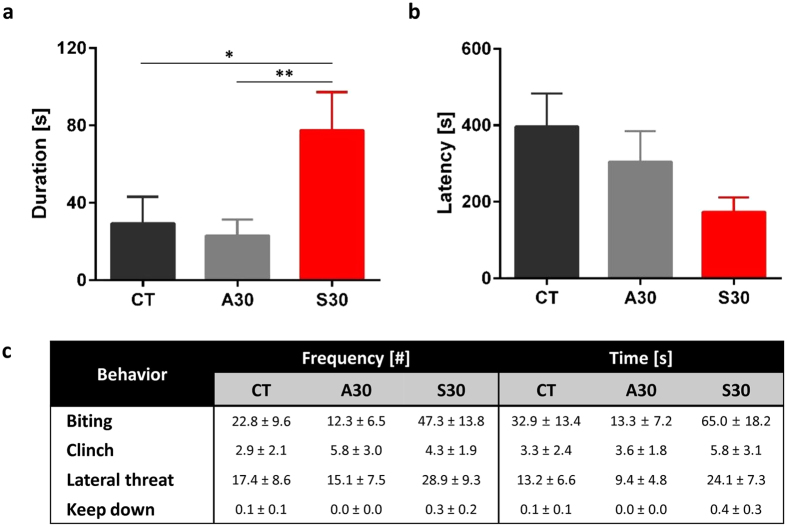
Aggressive behaviors in adult mice. (**a**) Latency ([s]; [seconds]) to show the time of first attack performed by resident mice against intruders following consumption of sucrose solution, aspartame solution, or plain water for 8-week. (**b**) Duration ([s]; [seconds]) to show the total time of all aggressive behaviors. (**c**) Frequency (the number of attacks) and time (total time of each aggressive behavior) of aggressive behaviors. All data are presented as mean ± S.E.M. P-values were generated by Mann-Whitney U test to determine significant difference in S30 compared with CT or A30. *p < 0.05, **p < 0.01, ***p < 0.001.

In order to examine if long-term consumption of a sucrose solution would alter aggressive behaviors, resident-intruder tests were conducted to evaluate male-male territorial aggression. We measured the frequencies and the durations of aggressive attacks by resident mice against the unfamiliar intruder mice, consisting of biting, clinch, lateral threat and keep down. There was a tendency towards increases in the time and the number of events for each behavior in the S30 compared with CT or A30 groups (Fig. 2c), albeit with no significance differences being found. It was notable that 100% of mice on S30 showed biting and lateral threat, while a smaller percent of mice on CT or A30 showed those behaviors (78% in CT and 56% in A30 for biting; 44% in CT and 78% in A30 for lateral threat). The total duration of all aggressive behaviors (Fig. 2a) and the latency to show the first attack (Fig. 2b) were determined as well. Mice in the S30 group showed a dramatically significant increase in the duration of aggressive behaviors. Taken together, adult aggressive behaviors were promoted by continuous consumption of sucrose solution accompanied with changes in aggression-related hormones.

### Transcriptome analyses revealed consumption of sucrose solution induced inflammatory responses in the brain

**Figure 3:**
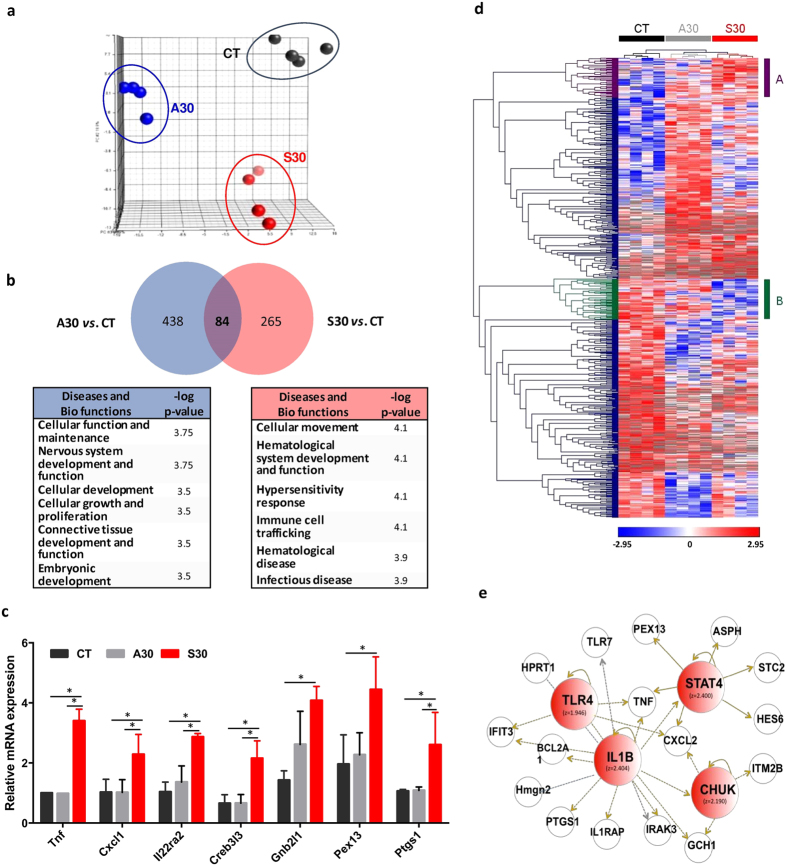
Transcriptome profiling analysis of hypothalamus in mice. (**a**) 3-D view of principal component analysis scores plot of brain transcriptome. Each spots indicates individual mouse in the group classified by different colors. Black spots, mice consumed tap water (control); Red spots, mice consumed sucrose solution (S30); Blue spots, mice consumed aspartame solution (A30). (**b**) Venn diagram summarizing genes that were differentially expressed (p < 0.05; fold-change >2.0) between S30 versus CT and A30 versus CT. Functional identification categorized by IPA; Top six categories of diseases and bio-functions were detected in S30 group compared to CT group except common genes with A30 versus CT (right) and in A30 group compared to CT group except common genes with S30 versus CT (left). Statistical significance was calculated by a right-tailed Fisher’s exact test in IPA and –log(p-value) is noted. (**c**) Relative mRNA expression including molecules associated with inflammation. (**d**) Hierarchical clustering and heat map of differentially expressed genes (>2-fold) in either S30 or A30 group compared to CT with two clusters; cluster A and cluster B. Red represents high relative expression and blue represents low relative expression of genes as shown in colored scale bar below the heat map. Clusters were established by normalized gene expression patterns as shown in gene expression view. Cluster A consists of genes that are only up-regulated (n = 68) in S30 compared to CT or A30; Cluster B is composed of down-regulated genes (n = 73) only in S30 compared to CT or A30. (**e**) Upstream regulator analysis in gene expression data of S30 mice compared with CT mice. Four significant molecules was identified by calculated z-score. Several genes and molecules as detected upstream regulators are involved in pro-inflammatory response (p = 4.40E-09) and inflammatory response (p = 1.60E-08) identified by IPA.

To develop molecular understandings of transcriptional networks that might contribute to the promoted aggression by sucrose consumption, we performed transcriptome analyses of brain. Four different biological replicates were randomly selected in each group and total RNAs from the hypothalamus of each mouse were applied to Illumina whole genome mouse arrays. 3,750 differentially expressed genes were identified by one-way ANOVA test with a cutoff of p-value < 0.05. Principal component analysis (PCA) showed that the gene expression profiles of the S30 group were readily distinguished from those of the CT or A30 groups (Fig. 3a). In the comparison of S30 *vs*. CT with the stringent thresholds of 2-fold changes and p < 0.05, we identified 522 sugar-responsive genes. A Venn diagram confirmed that the effects of sugar on the brain transcriptome were substantially different from those of the A30 group (Fig. 3b), since only 84 genes out of 349 significant genes in the S30 group overlapped with the set of aspartame-responsive genes. To identify the significant biological functions modulated by aspartame or sugar, aspartame-specific genes and sugar-specific genes were categorized based upon their functions and subsequently, the significance was tested with Fisher’s exact test (Fig. 3b). While the aspartame solution changed the expression of genes in categories related to development and growth, such as *Cellular Function and Maintenance, Nervous system development and Function, Cellular Development*, and *Cellular Growth and Proliferation*, sugar modulated biological categories of *Cellular Movement, Hematological System Development and Function, Hypersensitivity Response, Immune Cell Trafficking, Hematological Disease*, and *Infectious Disease*. As a point of interest, all top 6 significant biological functions affected by the sucrose solution are related, directly or indirectly, to inflammatory responses. It is notable that gene expression signatures of pro-inflammatory responses including cytokines such as TNF-α and CXCL1 were dramatically changed in S30 mice compared those of CT or A 30 mice (Fig. 3c). The observations that aggressive behaviors were enhanced by sucrose but not by aspartame prompted us to further analyze the differentially expressed genes specifically in S30 but not in A30. We therefore focused on the two clusters, A and B, from the hierarchical clustering analysis (Fig. 3d). In cluster A, normalized gene expression values were increased only in S30, whereas in cluster B, gene expression values were decreased only in S30. We confirmed that *Inflammatory Response* was still the top significant category in cluster A (p = 2.95E-03) and cluster B (p = 1.73E-03). In cluster A, inflammatory molecules such as *Mgst1, Ces3, Spn, Abcb4, Cxcl1, P2rx7*, and *Sh2b3* were included. In particular, *Cxcl1* plays a role in inflammation as a leukocytes chemoattractant. *Sh2b3* encodes a member of the SH2B adaptor family of proteins that are involved in a range of signaling activities activated by growth factors and cytokine receptors. Likewise, transcripts in cluster B associated with *Immunological Disorder* included *Serpinb3a, Tbxa2r, Tlr7, Slit2, Dusp10*, and *Pik3r1*. We next carried out further bioinformatic analysis to predict major upstream regulators of the genes in clusters A and B, since it would be critical to understand the upstream regulatory molecules and their associated mechanisms of action to provide biological insights into the observed changes in expression (Fig. 3e). The top four predicted upstream regulators ranked by Z-score were *IL1B* (z-score = 2.404), *STAT4* (z-score = 2.400), *CHUK* (z-score = 2.190), and *TLR4* (z-score = 1.946). All these regulators are well known contributing factors to the development of inflammatory responses. Together, long-term consumption of sucrose solution induced the activation of transcriptional networks related to pro-inflammatory responses in the brain.

### Heightened numbers of circulating inflammatory cells were found in sucrose consuming mice

**Figure 4:**
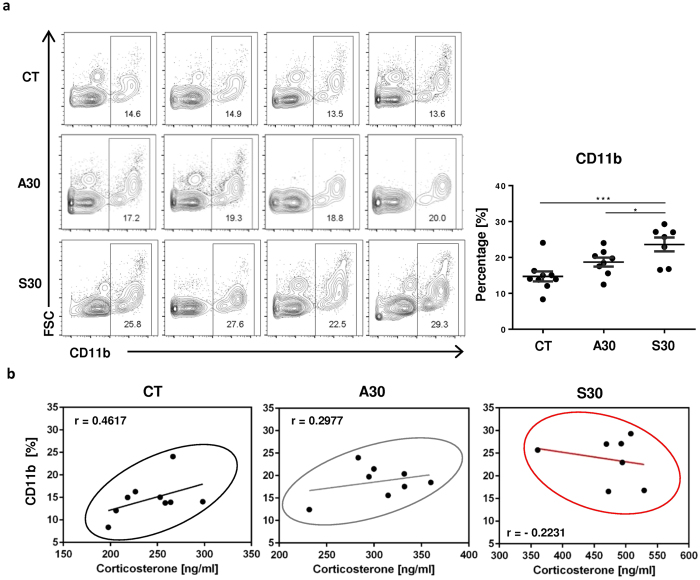
Flow cytometric analysis of myeloid lineage leukocytes in peripheral blood. (**a**) The effect of consuming either sucrose solution or aspartame solution during the juvenile period on the numbers of CD11b^+^ cells in peripheral blood (PBL) analyzed by flow cytometry. Representative flow cytometric data are displayed for each group. (**b**) Correlations between concentration of serum corticosterone and the percentage of CD11b cells in PBL for consuming plain water, aspartame solution, or sucrose solution in sequence. Each dot indicates each value of individual mouse and each solid line represents equation of correlation elicited from linear regression model.

The observations that consumption of a sucrose solution increased the serum levels of corticosterone and it activated pro-inflammatory transcriptional networks in the brain, led us to determine the number of inflammatory cells in the peripheral blood (PBL). Flow cytometric analyses were carried out to assess PBL CD11b^+^ myeloid lineage leukocytes in each mouse. The frequencies of CD11b^+^ cells were significantly higher in PBL of S30 mice than those of CT or A30 mice (Fig. 4a). Glucocorticoids normally reduce inflammatory responses by recruiting leukocytes to the sites of inflammation through glucocorticoid receptor signaling in those cells. However, in a pathological condition or under chronic stress, it fails to recruit those inflammatory cells as a result of developing glucocorticoid resistance^39^. Therefore, we investigated the associations between the percent of CD11b^+^ cells and corticosterone level using a linear regression model. Although the differences were not significant, the serum levels of corticosterone tended to be positively correlated with the percentages of CD11b^+^ cells in CT mice (r = 0.4617) and in A30 mice (r = 0.2977). On the contrary, in S30 mice, there was a pattern of negative correlation between the frequencies of CD11b^+^ cells and the levels of corticocosterone (r = −0.2231). These results imply that inflammatory cells in mice on S30 might respond differently to the hormone as compared to those on CT or A30. In summary, sucrose consumption increased the levels of corticosterone, but the hormone failed to suppress inflammatory responses.

## Discussion

To our knowledge, this is the first study investigating the effects of sucrose consumption on aggressive behavior in a controlled setting using animal models. First, we found a dramatic elevation in serum corticosterone levels in mice that consumed high sugar for a short interval of 2 weeks in a dose-dependent manner. This observation led us to examine the long-term effects of high sugar consumption that started in infancy and continued to adulthood. We found heightened levels of serum corticosterone when treated mice matured to adulthood as compared to control mice. Corticosterone, a dominant glucocorticoid in rodents that is comparable to cortisol in humans, is widely studied as a stress hormone and a well-established target in the search for hormonal modulators of social aggression^9^,^10^. In agreement with other studies, we observed that aggressive mice had higher levels of corticosterone. Several other hormones have also been reported for their associations with aggressive behavior. Local release of vasopressin within the lateral septum of the brains was reported during a resident-intruder test in adult male rats^7^. Vasopressin release correlated positively with inter-male aggression, and treatment with a vasopressin V1a receptor antagonist reduced the aggression of highly aggressive rats^8^. Oxytocin was reported to inhibit aggressive behaviors in a study showing that oxytocin injection into the brain decreased the frequency of biting in lactating female rats^4^. However, conflicting results were also reported in studies showing that oxytocin knockout (OT^−/−^) mice exhibited a significant decrease in the duration of aggressive attacks in a resident-intruder test compared with wild-type (OT^+/+^) control mice^6^. The present study revealed that there was no significant difference in expression of vasopressin in S30 mice as compared to CT or A30 mice. On the contrary, expression of oxytocin in S30 mice was significantly higher than in the other cohorts. In view of the reported conflicting roles of oxytocin in regulating aggressive behavior, our results demonstrate that social aggression promoted by SSB consumption might be mediated by corticosterone. It is also notable that ingestion of the artificial sweetener did not mimic the role of the sugar in elevating corticosterone levels or in promoting aggressive behaviors.

The consumption of a high sucrose solution resulted not only in hormonal changes but also in alterations in transcriptional networks in the brain. The hypothalamus is one of the regions in the central nervous system in which aggressive behaviors are regulated^40^,^41^. Although we used the entire hypothalamus for a transcriptome profiling analysis with the aim of elucidating global transcriptional networks of aggressive behavior (Fig. 2) as related to physiological functions-energy balance (Supplemental Fig. 1) and endocrine functions (Fig. 1), it is worth noting that the hypothalamus is a heterogeneous region consisting of several anatomical and functional subdivisions^42^. The precise location and particular neurons in the hypothalamus implicated in aggressive behaviors are actively being investigated. Recent studies identified ventromedial (VMN)^40^,^43^, premammillary nuclei (PMN)^40^,^43^ and anterior hypothalamic nuclei (AHi)^44^ as associated with aggressive behaviors. Through extensive bioinformatic analyses of the transcriptome in the hypothalamus, we found SSB consumption activated the transcriptional network of inflammatory responses with dramatic increases in the levels of TNFα and CXCL1. Furthermore, the frequency of CD11b^+^ cells in PBL was significantly higher in S30 mice than in CT or A30 mice. These circulating inflammatory cells tend to be poorly responsive to corticosterone compared to the cells in CT or A30 mice. The poor responsiveness of myeloid lineage leukocytes to the hormone has been previously reported in a human study wherein chronic stress resulted in failure to down-regulate the inflammatory response^39^. Furthermore, it has been reported that there is an association between aggressive behavior and elevated C-reactive protein levels in schizophrenic inpatients^45^, and elevated plasma inflammatory markers in individuals with intermittent explosive disorder^46^. We also observed serum proinflammatory cytokines such as IL6 and TNFa were slightly but not significantly higher in S30 group than those in CT or A30 (Supplemental Fig. 2). In addition, several studies showed that pro-inflammatory responses of the CNS following glucocorticoid stimulation regulated the immune system, contributing to altered behaviors^47^,^48^. That is, physiological stress induced the activation of the neuroimmune system with increases in neural pro-inflammatory cytokines and chemokines which facilitate peripheral immune cell trafficking to the brain, ultimately resulting in abnormal behavioral outcomes^49^. These results support our findings that the aggressive behavior shown in S30 mice is related to augmented neural inflammation accompanied by dysregulated immuno-modulating functions of corticosterone.

The behavioral outcomes shown in A30 mice were different from those of S30 mice, despite equivalent sweetness of the aspartame solution to that of the sucrose solution. This indicates that sugar-induced aggressive behavior is not due to the taste of sweetness. There was a report that acute high-dose administration of aspartame reduced aggressive attacks in rats^50^. However, others reported that rats receiving an aspartame solution would make longer and more numerous aggressive responses in association with reduced brain tryptophan levels and serotonin synthesis^51^. We observed that artificial sweetener consumption changed neither the levels of aggression hormones nor the transcript levels of genes involved in serotonin metabolism. Transcriptional profiles of brains from A30 mice turned out to be dissimilar to those in S30 mice. A number of aspartame-specific genes that were altered in their expression in response to aspartame but not to sugar are involved in the development and function of the nervous system. A study reported that aspartame produced a biphasic modulation of internal calcium that was associated with up-regulation of calcium in resting neurons and down-regulation in activated neurons^52^. In another report, aspartame-treated animals showed an increase in the expression of apoptotic genes and consequently, enhanced neuronal cell death^53^. These results suggest that aspartame plays distinct roles in neuronal functions compared to the roles played by sugar in the brain.

Many studies have reported that intake of sucrose solution can induce abnormal metabolic status, including high levels of blood triglyceride, glucose and insulin, leading to body weight gain in human^29^,^54^ and animal models^27^,^55^,^56^,^57^. However, in the present study, there were no significant changes in the levels of glucose, insulin, or triglycerides in the blood of S30 mice as compared to CT or A30 mice (Supplemental Fig. 1). Unexpectedly, their body weights were significantly lower than those of CT or A30 mice, although the evaluations of growth curve in S30 mice showed that they followed a normal growth pattern unlike that of mice on an early malnutrition diet^58^. In keeping with our results, other studies^59^,^60^,^61^,^62^ also reported no significant changes in body weight associated with consumption of sucrose. This discrepancy might be generated, at least in part, by the fact that different experimental designs, such as experimental diet, experimental period, and animal strains have been applied. The metabolic effects of sucrose consumption examined together with a high fat diet might be different from those associated with a normal fat diet^57^. In addition, the initiating time point in the life cycle and duration of the sucrose challenge might be important contributing factors^27^,^56^,^63^. Animal strain differences might also contribute to the discrepancy as well. It was notable that analysis of intestinal sucrase showed that the enzyme was less active in C57BL/6 mice than in other strains when the diet was high in sucrose^61^. Furthermore, according to a study that reported higher locomotor activity in mice fed a sucrose solution^55^, energy expenditure due to augmented activity could possibly have contributed to reduced body weight gain and other metabolic indices, such as blood glucose or triglycerides, in mice with sucrose consumption. It is well known that glucocorticoids, in addition to functions in the CNS, have a wide range of regulatory roles not only in immune functions but also in catabolic functions in energy metabolism. In this regard, a number of studies report a link between reduced body weight and aggression. Reduction in body weight has been positively correlated to altered mood^12^ and hostile aggression^11^ in both adults and female adolescents^13^. In addition, chronically stressed mice showed high levels of corticosterone and, consequently, reduced body weight leading to enhanced behavioral invigoration^14^.

In conclusion, the present studies showed the promoting effects of long-term sucrose solution consumption on aggressive behaviors. The promoting effects were mediated by changes in serum corticosterone and body weight. The roles played by sugar in social aggression were not related to the sweet flavor, since they were clearly distinct from those played by an artificial sweetener. Further, transcriptome analyses provided molecular understandings of the underlying mechanisms by which sugar drinks induced aggressive behaviors, and showed that long-term sugar drink consumption dysregulated the transcriptional networks of inflammatory responses in the brain. Accordingly, heightened numbers of inflammatory cells in the peripheral blood were found in mice fed the sucrose solution. These results demonstrate that long-term sugar consumption from infancy to adulthood promotes aggressive behaviors and that these effects are modulated by dysregulation of inflammatory responses in the brain. Therefore, in addition to the known benefits of sugar reduction on lowering the risk for physical diseases and disorders, a reduction in sugar intake might assist in resolving social problems related to aggressive behaviors.

## Methods

### Mice

3 week-old male C57BL/6J mice after weaning were obtained from Jackson Laboratory (Bar Harbor, Maine, USA) and were acclimated for 4 days. Experimental mice were assigned to three groups (8–9 mice per each group) and kept in individual plastic cages and maintained on a daily 12 h/12 h light cycle with 60% relative humidity under conditions of the animal facility at the College of Veterinary Medicine of Seoul National University (Seoul, South Korea). Single housing was necessary since the aggressive behavior test requires that mice be kept isolated from other mice. Each group was provided a normal diet composed of 20 kcal% protein, 70 kcal% carbohydrate and 10 kcal% fat (3.85 kcal/g, D12450J, Research diets) with drinks composed of 30 w/v% sucrose solution (Saccharose, Daejung, South Korea), aspartame solution (Aspartame 100%, The Nutrasweet Company, USA) with equivalent sweetness of 30 w/v% sucrose solution (aspartame is about 200 times sweeter than sugar according to U.S. Food and Drug Administration) or plain water ad libitum for 8 weeks. On the 56th day after overnight fasting, mice were anesthetized and sacrificed through intraperitoneal injection of 20% urethane (U2500, Sigma-Aldrich, USA) after behavior test. The mice were maintained according to the guidelines of Seoul National University Animal Experiment Ethics Committee. All animal experiments were performed after receiving approval of the Institutional Animal Care and Use Committee of the Institute of Laboratory Animal Resources, Seoul National University (Institutional Animal Care and Use Committee permit number: SNU-140129-6). All experiments were carried out in accordance with the guidelines and regulations.

### Resident-Intruder test

On the 52th day, the resident-intruder test was performed to measure and evaluate inter-male aggression in adult mice. Resident mice (8–9 mice per each group) were housed individually to establish their own territory in a home cage. The intruder mice were group-housed males, 4 weeks younger than the resident mice. On day 50, each intruder mouse was introduced to a resident mouse in the resident’s home cage temporarily divided by an acrylic board with several holes. At first, the resident and intruder could experience only by their scent through the holes in acrylic board for 5 minutes in order to inform the resident that there is a stranger in its home cage. Thereafter, the acrylic board was removed and they could explore each other for 10 minutes. All processes of the resident-intruder test were recorded. Latency to first attack, duration and frequency of following aggressive behaviors were measured and averaged by three different researchers; biting, clinch, lateral threat, keep down.

### Serum ELISA

Blood samples of mice were collected by heart puncture under 20% urethane anesthesia on the day of sacrifice between 9 am to 11 am. Serum was taken from blood samples centrifuged at 1,500 × g for 20 min and stored at −80 °C. Serum corticosterone levels were measured using Corticosterone ELISA kit (Abcam LLC, Cambridge, UK) in accordance with manufacturer’s instructions. The serum concentrations of TNFα were measured using a commercial ELISA kit (Biolegend); a 100 μl serum sample and assay diluent were placed in each well of a 96-well plate coated with a monoclonal mouse IgG against TNFα. The mixtures were incubated for 2 h at 4 °C, followed by aspiration and three washes with washing buffer. Subsequently, 200 μl conjugate solution was placed into each well and incubated for 2 h at room temperature. After washing each well four times with washing buffer, 200 μl of substrate solution prepared with equal amounts of stabilized hydrogen peroxide and tetramethylbenzidine was added for a 20 min reaction in the dark. The reaction was quenched by adding 50 μl stop solution (2NH_2_SO_4_)^64^.

### Real-Time PCR (qPCR)

Mouse brains were obtained and each hypothalamus was separated and snap frozen in liquid nitrogen. RNA was extracted and purified from deep-frozen hypothalami of brain samples using RNAqueous-4PCR kit (Ambion, Austin, TX, USA). Synthesis of cDNA was done by MessegeSensor™ RT kit (Ambion, Austin, TX, USA). Detection of transcripts of vasopressin (NCBI Reference Sequence: NM_009732.1) and oxytocin (NCBI Reference Sequence: NM_011025.4) was performed using Applied Biosystems^®^ SYBR^®^ Green PCR Master Mix and specific primer pairs amplifying vasopressin (forward primer: 5′-CCAGGATGCTCAACACTACG-3′, reverse primer: 5′-CTCTTGGGCAGTTCTGGAAG-3′) and oxytocin (forward primer: 5′-CCTACAGCGGATCTCAGACTGA-3′, reverse primer: 5′-TCAGAGCCAGTAAGCCAAGCA-3′) respectively. Each gene expression level was normalized to GAPDH (forward primer: 5′-TGCACCACCAACTGCTTAG-3′, reverse primer: 5′-GATGCAGGGATGATGTTC-3′).

### Microarray hybridization

For microarray hybridization, total RNA was isolated by homogenizing tissue samples of hypothalami using TissueLyser II (QIAGEN, Crawley, UK) and purified using a DNA-free RNA isolation kit (RNAqueous-4PCR kit; Ambion, Austin, TX, USA) according to the manufacturer’s instructions. The integrity and quantity of total RNA were assessed with a NanoDrop 2000 Spectrophotometer (Thermo Fisher Scientific, Wilmington, DE, USA) before the microarray experiments. Its quality was assessed by agarose gel electrophoresis using 1% gels. RNA samples were first amplified for array analysis using the Illumina TotalPrep RNA Amplification Kit (Ambion, Austin, TX, USA) in accordance with the manufacturer’s instructions. Briefly, 500 ng of total RNA was used to prepare labelled cRNA with overnight incubation according to the manufacturer’s protocol. The quality and quantity of the labelled cRNA were monitored using a NanoDrop 2000 Spectrophotometer (Thermo Fisher Scientific, Wilmington, DE). Amplified cRNA (1.5 ng) was hybridized on MouseWG-6 v2 Expression BeadChip arrays, containing more than 45,200 well-annotated Ref transcripts in mice, according to the manufacturer’s standard protocol. The array were then scanned on a BeadArray Reader (BeadStation 500 G Instrument, Illumina Inc.), and Spot images were identified and quantified by the Genome Studio software v1.0.2. (Illumina Inc.).

### Identification of significant genes

The raw data from microarray were pre-processed through three steps: background correction was performed, the data were then log-transformed to log2 scale, and normalized by the quantile normalization method implemented in the Genome Studio software (Illumina Inc.). Significant differences among three groups were identified using ANOVA test (p < 0.05) on log2-transformed normalized intensities by Partek^®^ Genomics Suite software v6.3 (Partek, St Louis, MI) (http://www.partek.com/partekgs). Transcripts with more than a 2-fold differential were selected for each specific comparison analyzed.

### Bioinformatic analysis of gene expression data

Principal component analysis (PCA) was carried out by Partek^®^ Genomics Suite software v6.3 (Partek, St Louis, MI) (http://www.partek.com/partekgs). Hierarchical clustering analysis was performed with Genesis software v1.7.5^65^ using the Pearson correlation distance matrix with average linkage algorithm. Functional identification was categorized by the use of QIAGEN’s Ingenuity^®^ Pathway Analysis (IPA^®^, QIAGEN Redwood City, www.qiagen.com/ingenuity). Upstream regulator analysis was performed to predict upstream regulators with a calculated z-score with indirect or direct relationships in the dataset through IPA.

### Flow cytometry (FACS) analyses

Fresh peripheral blood lymphocytes (PBLs) were prepared on day 56, by incubating blood with ACK (ammonium-chloride-potassium) buffer to lyse red blood cells at room temperature for 3–5 minutes, and stained using FACS buffer (1X phosphate-buffered saline [PBS] with 0.1% bovine calf serum and 0.05% sodium azide) containing phycoerythrin (PE)-conjugated mAb to CD11b (M1/70, eBioscience) at 4 °C for 30 minutes. After washing with FACS buffer, the cells were analyzed by a FACSCalibur (BD Bioscience, Franklin Lakes, NJ, USA) and Flowjo software (Tree star, Ashland, OR, USA)^66^.

### Statistical Analysis

All data were expressed as the mean ± S.E.M. Statistical significance (p-value < 0.05) was evaluated by Mann-Whitney U test. Statistical analyses were performed using Graph Pad Prism 5 software (GraphPad Software Inc., La Jolla, CA, USA) and SAS Enterprise Guide 6.1 (SAS Institute Inc., Cary, NC, USA). For gene analyses, significances for functional enrichment of specific genes were determined by a right-tailed Fisher’s exact test as the negative log of the probability that the number of focus genes is not due to random chance.

## Additional Information

**How to cite this article:** Choi, J.-Y. *et al*. Long-term consumption of sugar-sweetened beverage during the growth period promotes social aggression in adult mice with proinflammatory responses in the brain. *Sci. Rep.* **7**, 45693; doi: 10.1038/srep45693 (2017).

**Publisher's note:** Springer Nature remains neutral with regard to jurisdictional claims in published maps and institutional affiliations.

## Supplementary Material

Supplementary Information
