# Supplementary material for: Long-term consumption of sugar-sweetened beverage during the growth period promotes social aggression in adult mice with proinflammatory responses in the brain
Source: Sci Rep. 2017 Apr 10;7:45693. doi: 10.1038/srep45693 (PMC5385878; doi:10.1038/srep45693)
Supplement: Supplementary Information [file srep45693-s1.pdf]

Long-term consumption of sugar-sweetened beverage during the growth period promotes social aggression in adult mice with proinflammatory responses in the brain

Jung-Yun Choi*[1], *Mi-Na Park*[1], *Chong-Su Kim*[1], *Young-Kwan Lee*[2], *Eun-Young Choi*[2], *Woo Young Chun*[3], *Dong-Mi Shin*[1,4]*

[1]Department of Food and Nutrition, Seoul National University, Seoul 08826, Korea

[2]Department of Biomedical Sciences, Graduate School of Seoul National University, Seoul 03080, Korea

[3]Department of Psychology, Chungnam National University, Daejeon 34134, Korea.

[4]Research Institution of Human Ecology, Seoul National University, Seoul 08826, Korea

*corresponding authors: shindm@snu.ac.kr

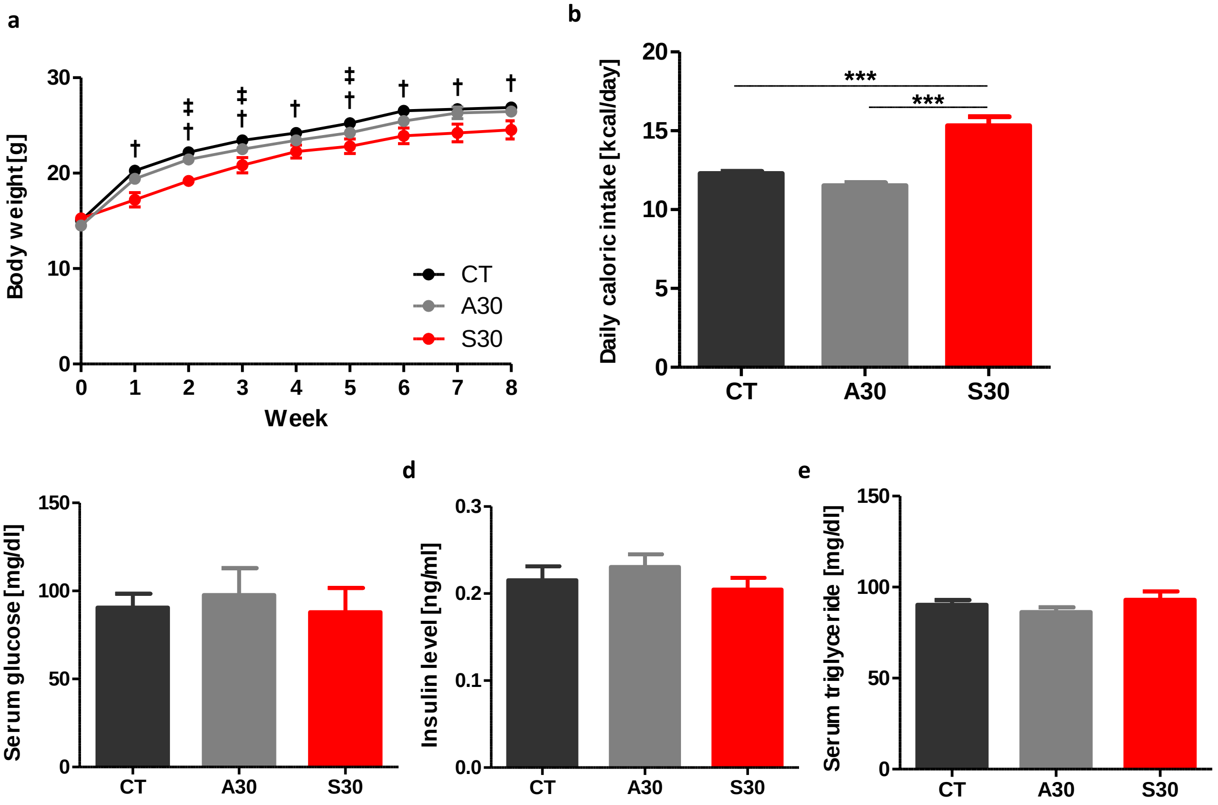

Supplementary Figure 1 | Metabolic parameters in mice following consumption of plain water, aspartame solution or sucrose solution. (**a**) Body weight of mice in all group (n = 8-9 per each group) gradually increased until the end of experimental period (8-week) regardless of kinds of solution consumed in each group. Statistical significance was presented; CT versus. S30 († $p < 0.05$); A30 versus. S30 (‡ $p < 0.05$). (**b**) Daily caloric intake in mice following consumption of each drinks. Daily caloric intake was calculated based on diet and drink intake. (**c**) Serum glucose levels, (**d**) Concentrations of serum insulin and (**e**) Serum triglyceride levels in mice consuming plain water, aspartame solution or sucrose solution from infancy to adulthood. All data are presented as mean ± S.E.M. Mann-Whitney U test was performed to determine significant difference in S30 compared with CT or A30. * $p < 0.05$, ** $p < 0.01$, *** $p < 0.001$.

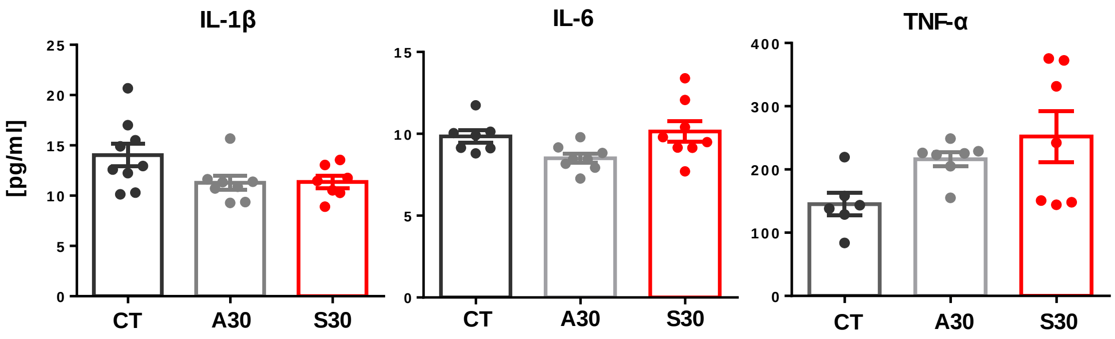

Supplementary Figure 2 | The serum levels of IL1b, IL6 and TNF-α (tumor necrosis factor-α). Amount of proteins were determined by ELISA n serum in mice following each solution; plain water, aspartame solution, or sucrose solution. Each dot indicates individual mouse.
